# Causality Analysis of Google Trends and Dengue Incidence in Bandung, Indonesia With Linkage of Digital Data Modeling: Longitudinal Observational Study

**DOI:** 10.2196/17633

**Published:** 2020-07-24

**Authors:** Muhammad Syamsuddin, Muhammad Fakhruddin, Jane Theresa Marlen Sahetapy-Engel, Edy Soewono

**Affiliations:** 1 Faculty of Mathematics and Natural Sciences Institut Teknologi Bandung Bandung Indonesia

**Keywords:** dengue, Google Trends, infodemiology, infoveillance, vector error correction model, Granger causality

## Abstract

**Background:**

The popularity of dengue can be inferred from Google Trends that summarizes Google searches of related topics. Both the disease and its Google Trends have a similar source of causation in the dengue virus, leading us to hypothesize that dengue incidence and Google Trends results have a long-run equilibrium.

**Objective:**

This research aimed to investigate the properties of this long-run equilibrium in the hope of using the information derived from Google Trends for the early detection of upcoming dengue outbreaks.

**Methods:**

This research used the cointegration method to assess a long-run equilibrium between dengue incidence and Google Trends results. The long-run equilibrium was characterized by their linear combination that generated a stationary process. The Dickey-Fuller test was adopted to check the stationarity of the processes. An error correction model (ECM) was then adopted to measure deviations from the long-run equilibrium to examine the short-term and long-term effects. The resulting models were used to determine the Granger causality between the two processes. Additional information about the two processes was obtained by examining the impulse response function and variance decomposition.

**Results:**

The Dickey-Fuller test supported an implicit null hypothesis that the dengue incidence and Google Trends results are nonstationary processes (*P*=.01). A further test showed that the processes were cointegrated (*P*=.01), indicating that their particular linear combination is a stationary process. These results permitted us to construct ECMs. The model showed the direction of causality of the two processes, indicating that Google Trends results will Granger-cause dengue incidence (not in the reverse order).

**Conclusions:**

Various hypothesis testing results in this research concluded that Google Trends results can be used as an initial indicator of upcoming dengue outbreaks.

## Introduction

Dengue is known as an infectious disease, which is caused by the dengue virus from *Flaviviridae* and genus *Flavivirus* families. This virus has four serotypes, namely DEN-1, DEN-2, DEN-3, and DEN-4 [1-3]. Infection by one of these four serotypes does not give cross-protective immunity. Hence, people who live in endemic areas can be reinfected by the other three serotypes throughout their lifetime [4]. *Aedes aegypti* and *Aedes albopictus* mosquitoes are vector transmitters of dengue. The disease is transmitted by mosquitoes through arthropod vectors in tropical and subtropical areas around the world [5]. As the most rapidly spreading mosquito-borne disease in the world, dengue fever has affected the lives of approximately 1.8 billion people in Southeast Asia alone. In the dengue-endemic region, Indonesia is one of the largest countries, with a population of 267 million [6]. Since the first dengue incidents reported in 1968, the number and range of dengue incidents in Indonesia have increased nationwide [7]. All 34 provinces in Indonesia have been reported to have dengue cases, showing the extensive range of the disease [8]. Early detection of disease activity can reduce the impact of the disease [9].

Bandung is one of the crowded cities in Indonesia. It has the highest dengue incidence, especially in West Java. Daily habits, landscape structures, weather, and the ecosystem in the city play roles in dengue vector breeding as primary factors for dengue transmission. The climate in Bandung is a mountainous climate (humid and cold), with an average temperature of 23.5°C. The average rainfall is 200.4 mm, and there are on average 21.3 rainy days per month. It is an ideal environment for *Aedes aegypti*. According to the Extraordinary Early Childhood Awareness System (SKDKLB-DBD) report, Bandung had the highest dengue incidence from 2002 to 2006, with a total of 22,335 infected people. In January 2019, the West Java Provincial Health Office recorded 236 dengue cases in Bandung. For these reasons, we selected Bandung as our study area to investigate and analyze the association between dengue data from Google Trends and dengue incidence data from a reputable hospital in Bandung.

In this modern world, it is impossible to say that technology, especially the internet, does not influence human lives. Over the years, research has been performed to investigate the accuracy of using internet search engine data to predict real-life phenomena, such as influenza epidemics and flu trends [9], stock markets [10-12], house prices [13,14], and tourism demand [15-17]. Google Trends is a public website belonging to Google Inc that offers data based on Google Search, which shows how frequently a particular search term is entered.

According to StatCounter, in 2016, Google was the most used text search engine in Indonesia. About 97% of people who use the internet in Indonesia use Google. It is assumed that Indonesian people show the trends to find information about dengue on the internet [18]. Therefore, we hypothesized that the popularity of dengue on Google has a correlation with the dengue incidence in Indonesia. Several studies about Google Trends results and the relationship with various diseases have been carried out, such as a study on dengue fever in Indonesia by using moving average analysis [19]. Other Google Trends–related work is presented in the following sentences. Dengue in several countries (Bolivia, Brazil, India, Indonesia, and Singapore) has been studied to maximize a fitting model by using a univariate linear model [20]. A spatiotemporal analysis of dengue incidence has been performed by using an exponential generalized autoregressive condition heteroscedastic model [21]. In other studies, we can see a web-based search for the early identification of the disease prevalence of coronary heart disease [22], forecasting of influenza cases using internet data [23], the use of Google Trends in health care research by using correlation analysis [24], infodemiology and infoveillance [25-27], a framework of social media data and quality assessment for a reporting standard [28], the spread of pertussis in Europe [29], and the spread of AIDS in the United States [30]. This led us to hypothesize that dengue incidence and Google Trends results have a long-run equilibrium.

We started our research with an initial hypothesis that the popularity of dengue on Google correlates with dengue cases in Bandung. We then investigated the relationship between these two data by using the Dicky-Fuller test, error correction model (ECM), impulse response function, and variance decomposition. We hoped that information from Google Trends can be used for the early detection of upcoming dengue outbreaks so that policymakers can prepare for the early prevention or control of the epidemic.

## Methods

### Collected Data

Google Trends is a website that analyzes the popularity of a topic in various countries and various languages based on search requests. The data source is over the internet and open source and can be easily accessed by everyone. In Google Trends, a user can enter a keyword in the form of words or phrases related to the selected topic or cases. Google Trends is not case sensitive but takes into account spelling errors that might occur. Users can specify the duration of time they want to review by selecting a time range or specifying a date. In addition, users can specify the area to be reviewed by selecting the appropriate country, city, or province or state. They can also see the popularity of these keywords globally by selecting the option *worldwide*.

**Figure 1:**
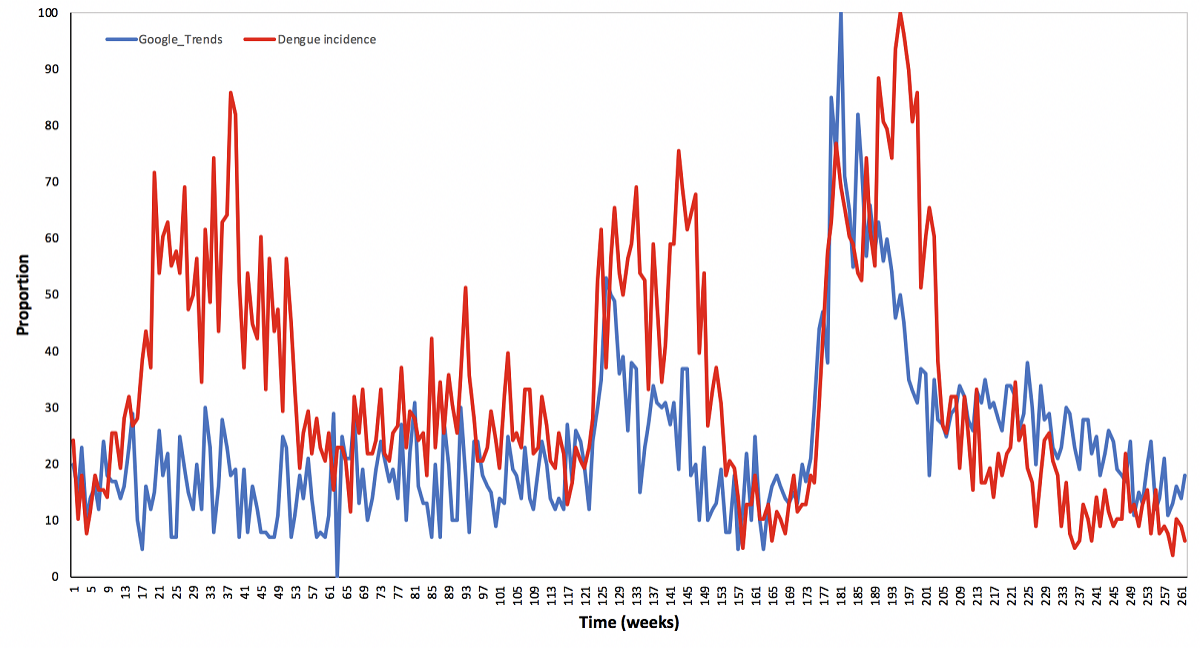
Dengue data plot from Google Trends and reported cases in Bandung.

Data used in this study are time-series data of dengue incidence from Santo Borromeus Hospital in Bandung, as well as popularity data taken from Google Trends via the website (Figure 1). The time range used in this paper is from September 9, 2012 until September 7, 2017. We consider weekly data over the time interval, and there were 261 data points. We assumed that the incidence data represent all dengue cases in Bandung owing to the location of the hospital in the city center. Google Trends data were obtained from the Google Trends website on google [31] by entering the keyword “demam berdarah dengue” plus “dbd.” With regard to the technique of taking data, we followed previous guidelines [32] when using Google Trends for valid results in our study by selecting the appropriate keyword(s), region(s), period, and category. Quotation marks were used so that the search results only showed the popularity of keywords in that exact order. The data represented the popularity of the keyword “demam berdarah dengue” plus “dbd” found on Google. Google Trends normalizes its popularity data by dividing each data point by the total amount of searches at a given time and location. This results in a proportion scaled in a range from 0 to 100. This scale shows the popularity of specific keywords relative to the time and location of the query.

### Stationary Test

We performed a stationary test for the time series data of Google Trends (*X_t_*) and the dengue incidence (*Y_t_*). A time series {*y_t_*} is said to be stationary if it satisfies the following conditions: (1) *E*[*y_t_*] = µ<∞; (2) *Var*[*y_t_*] = σ^2^<∞; and (3) *Cov*[*y_t_*, *y_t+s_*] = *γ_s_*<∞, for s>0.

Differencing a series produces another set of observations, such as the first differenced values, where △*y_t_* = *y_t_* − *y_t−1_*. Generalizing this operation and performing the difference operation as much as *n* times can be written as △^n^*y_t_* = *y_t_* − *y_t−n_*. If a series is stationary without any differencing, it is said to be integrated of the order 0 or *I*(0). However, if it is stationary only after differencing once, it is said to be integrated of the order 1 or *I*(1). The Dickey-Fuller test was used to detect the presence of a unit root and determine the stationarity of Google Trends and dengue fever incidence series.

### Cointegration Test

For cointegration, Engle and Granger [33] used the stationarity test of the residual series obtained from the long-run equilibrium equation. If the residual series, denoted by {*e_t_*}, is stationary, given that *y_t_* and *z_t_* are first-order stationary, they are cointegrated.

After finding the Google Trends and dengue incidence series to be first-order difference stationary, the long-run equilibrium relationship can be stated in the following form:

*Y_t_* = *β_0_* + *β_1_X_t_* + *e_t_* (**1**)

where *e_t_* denotes the residual.

Let {*ê_t_*} be the residual sequence. The series {*ê_t_*} contains the estimated values of deviations from the long-run relationship. By using the Dickey-Fuller test to check its stationarity, it was found that the level values of {*ê_t_*} were stationary.

### Estimation and Analysis of a Vector ECM

After a cointegrating relationship has been established, an ECM can be built to establish the short-run relationship between two variables. A likelihood ratio test can be used to determine the time lag of the vector ECM or the value of *p*. The regression equation for an ECM is as follows:

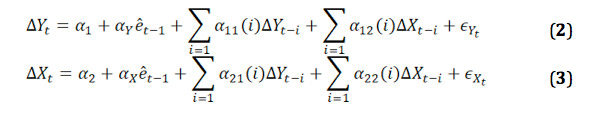



Analysis of cointegration shows that Google Trends and dengue incidence have a long-run equilibrium relationship. However, they are in disequilibrium in the short term. View equations 2 and 3 as a vector autoregression (VAR) model as follows:

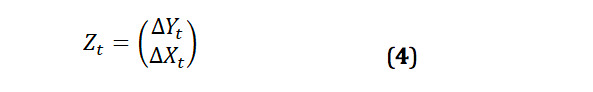



Hence, the vector ECM at hand can be written as a VAR model as follows:

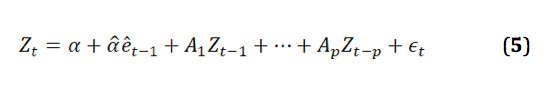



Before estimating the vector ECM, the optimal lag order is first determined.

### Causal Relationship Between Google Trends and Dengue Incidence

One way to test causality is to see whether the time lag of one variable is relevant for another variable. In a two-equation system with stationary variables *y_t_* and *z_t_* with *p* lags, it is said that {*y_t_*} does not Granger-cause {*z_t_*} if and only if the coefficients of *y_t_* in the equation for *z_t_* are equal to zero. In other words, if {*y_t_*} does not provide improvement for the forecasting performance of {*z_t_*}, {*y_t_*} does not Granger-cause {*z_t_*}. Granger causality only shows the effects of past values of {*y_t_*} toward the current values of {*z_t_*}.

In order to test Granger causality, a standard *F* test of the restriction *a*_21_(1) = *a*_21_(2) = … = *a*_21_(*p*) = 0 is performed.

In a cointegrated system, *X_t_* does not Granger-cause *Y_t_* if the values of Δ*X_t−i_* do not enter in the equation of Δ*Y_t_* and if *Y_t_* does not respond to deviation from the long-run equilibrium.

### Impulse Response Function and Variance Decomposition

To analyze the dynamic effects of the model in response to shocks and the effects on the two variables, the impulse response function and variance decomposition were examined.

## Results

### Stationary Test

**Figure 2:**
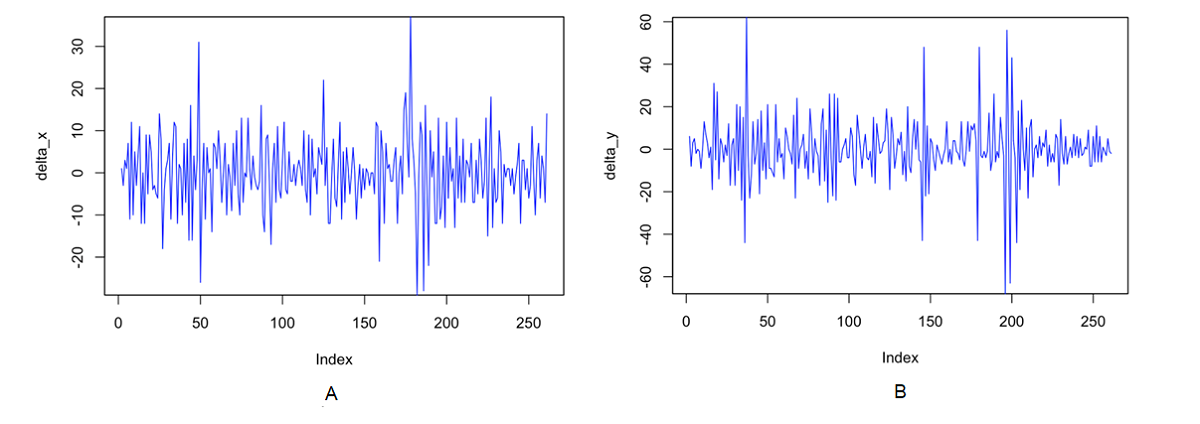
(A) *{X_t_}* and (B) *{Y_t_}* plots with one-time difference. X_t_: Google Trends data; Y_t_: dengue incidence data.

The stationary test results can be seen in Table 1, Figure 2A, and Figure 2B. The table shows that the level values of the Google Trends and dengue incidence series were nonstationary. However, Google Trends and dengue incidence data were found to be stationary after being differentiated once. This was done to reduce the fluctuations in the data.

**Table 1:** Dickey-Fuller test for Google Trends data, dengue incidence data, first differenced Google Trends data, and first differenced dengue incidence data.

Variable	Dickey-Fuller test statistic (value)	Dickey-Fuller critical value (N=250)
*X*_t_^a^	−2.42 (.02)	−2.58
*Y*_t_^b^	−2.24 (.03)	−2.58
*ΔX*_t_^c^	−21.76 (.01)	−2.58
*ΔY*_t_^d^	−27.85 (.01)	−2.58

^a^Google Trends data.

^b^Dengue incidence data.

^c^First differenced Google Trends data.

^d^First differenced dengue incidence data.

### Cointegration Test

The cointegration test results of ordinary least squares regression yielded that the long-run equilibrium relationship can be shown as follows:

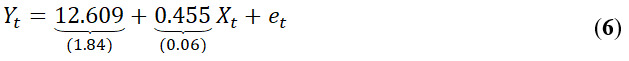



with *e_t_* denoting the residual.

Let {*ê_t_*} be the residual sequence. The series {*ê_t_*} contains the estimated values of deviations from the long-run relationship. By using the Dickey-Fuller test to check its stationarity, it was found that the level values of {*ê_t_*} were stationary. The results are shown in Table 2.

From previous results, it was seen that {*X_t_*} and {*Y_t_*} are *I*(1), and because the {*ê_t_*} series is stationary,{*X_t_*} and {*Y_t_*} are cointegrated. Hence, a vector ECM can be constructed.

**Table 2:** Dickey-Fuller test for the residual sequence.

Variable	Dickey-Fuller test statistic	Dickey-Fuller critical value (N=250)
*e*_t_^a^	−8.77	−2.58

^a^residual estimated as follows: *e_t_* = *Y_t_* − 12.609 − 0.455*X_t_*

### Likelihood Ratio Test to Find the Time Lag

The longest feasible lag length was set as 8 weeks. Thereafter, the value of the determinant of the variance-covariance matrix of a model with lag length eight was examined (denoted as Σ_8_) and compared with that of a model with lag length seven (denoted as Σ_7_). The likelihood ratio is (*T* − *c*)(lnΣ_7_ − lnΣ_8_), where *T* is the number of observations and *c* is the number of parameters that are estimated in each equation of the unrestricted system. In the case of comparing the eight-lag model to the seven-lag model, the value of *c* is 1 + 8*n*, with *n* being the number of variables, which is two in this case. If the likelihood ratio is smaller than the critical value (*χ*^2^_4_ at a significance of α=1%), the null hypothesis of the restriction *A*_8_=0 is rejected. This is done until lag 1.

The results of this test are shown in Table 3. The likelihood ratio test showed that the optimal number of lags needed for this vector ECM is three.

**Table 3:** Likelihood ratio test for lag length.

Number	*H*_0_	*H*_1_	Likelihood ratio	*χ^2^_4_*	Verdict
1	*A*_8_=0	*A*_8_≠0	7.655	13.277	*H*_0_ rejected
2	*A*_7_=0	*A*_7_≠0|*A*_8_=0	3.291	13.277	*H*_0_ rejected
3	*A*_6_=0	*A*_6_≠0|*A*_8_=*A*_7_=0	0.221	13.277	*H*_0_ rejected
4	*A*_5_=0	*A*_5_≠0|*A*_8_=…*A*_6_=0	2.543	13.277	*H*_0_ rejected
5	*A*_4_=0	*A*_4_≠0|*A*_8_=…*A*_5_=0	6.191	13.277	*H*_0_ rejected
6	*A*_3_=0	*A*_3_≠0|*A*_8_=…*A*_4_=0	19.666	13.277	*H*_0_ rejected
7	*A*_2_=0	*A*_2_≠0|*A*_8_=…*A*_3_=0	27.887	13.277	*H*_0_ rejected
8	*A*_1_=0	*A*_1_≠0|*A*_8_=…*A*_2_=0	60.361	13.277	*H*_0_ rejected

### Estimation of the ECM

After finding the optimal number of lags, an ECM model was built. The estimated vector ECM is as follows:

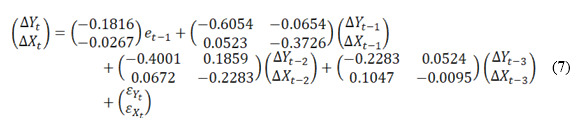



From the equation, it is seen that the speed of the adjustment parameter is −0.1816 for {*Y_t_*} and −0.0267 for {*X_t_*}. This means that when there is a deviation of 1 from the long-run equilibrium in the period *t* − 1, the number of dengue incidences will decrease by 0.1816 and dengue popularity in Google will decrease by 0.0267.

The speed of adjustment parameter for dengue incidence was nine times larger than the value for Google Trends, meaning that dengue incidence is more responsive to deviations from the long-run equilibrium. On the other hand, Google Trends only responds slightly to the aforementioned deviation.

It was found that this model has an R-squared value of 0.4128 for the Δ*X_t_* equation and 0.1511 for the Δ*Y_t_* equation, as well as an Akaike Information Criterion (AIC) value of 2370.2. Since the R-squared value is quite low, it can be said that the model cannot explain the data at hand accurately.

### Causal Relationship Between Google Trends and Dengue Incidence

Based on the vector ECM in equation 7, Granger causality was tested between Google Trends and dengue incidence. It was noted that at lag 2 and 3, *X_t_* Granger-causes *Y_t_* at a significance of α=5%, since the *P* obtained was similar (.04). However, *Y_t_* does not Granger-cause *X_t_*. This means that information from past values of Google Trends regarding dengue at a lag of 2 and 3 weeks is useful for explaining the present value of dengue incidence. The results are shown in Textbox 1.

**Granger causality test for Google Trends data and dengue incidence data.**

**Part 1: Does Google Trends Granger-cause incidence?**

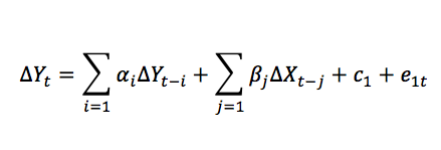

*H*_0_: *β*_1_=…=*β_j_*=0 (Google Trends does not Granger-cause incidence)

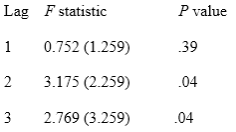

**Part 2: Does incidence Granger-cause Google Trends?**

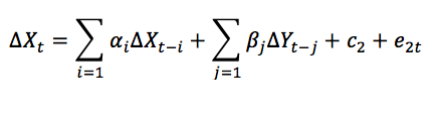

*H*_0_: *β*_1_=…=*β_j_*=0 (incidence does not Granger-cause Google Trends)

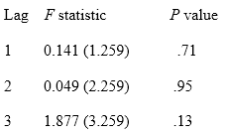

### Impulse Response Function and Variance Decomposition

The results for 12 periods (3 months) are obtained as presented below.

#### Impulse Response Function

**Figure 3:**
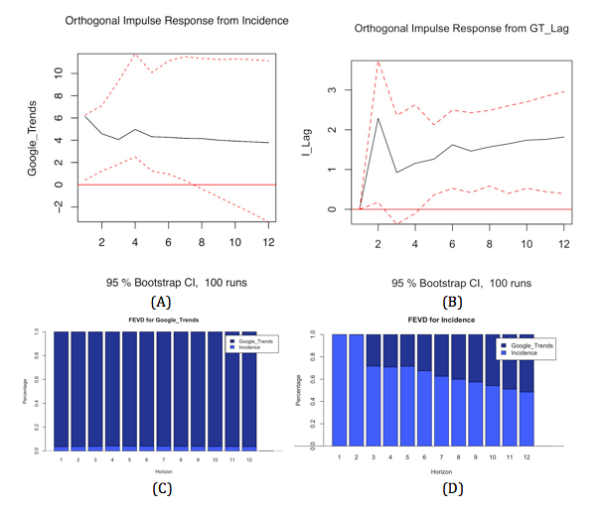
Impulse response function of (A) dengue data from Google Trends with respect to reported cases and (B) reported cases with respect to dengue data from Google Trends. Forecast error variance decomposition of (C) dengue data from Google Trends and (D) reported cases.

As shown in Figure 3B, a positive shock in dengue popularity on Google Trends has a relevant impact on dengue incidence. Dengue incidence shows a large increase after two periods. Thereafter, it shows a slight decrease, but then, it increases again slowly. Its effects seem to be long term, since the incidence keeps increasing until the end of the 12 periods. This phenomenon suggests that shocks in dengue fever popularity on Google have a relevant impact on dengue fever incidence.

On the other hand, through analysis of the response of dengue incidence to a positive shock, it was found that dengue popularity increases slightly and then remains constant. This behavior is presented in Figure 3A. After a positive shock in dengue incidence, there is little fluctuation in its popularity on Google Trends. This suggests that shocks in dengue incidence do not have a relevant effect on its popularity on Google Trends. Its effects are only short term and do not remain in the long run.

Generally, the impulse response function shows that Google Trends has a relevant impact on dengue fever incidence and has a long-term effect. On the contrary, dengue incidence has only a short-term and small effect on the popularity of dengue on Google.

#### Variance Decomposition

Variance decomposition estimates the contribution of shocks in a variable toward the response of another variable. As shown in Figure 3D, the contribution of dengue incidence to its variance gradually declines in the first two periods. Thereafter, it declines further until the contribution of dengue incidence is finally only around 40%. In the first period, Google Trends only has a small contribution to dengue incidence variance with only 0%. Thereafter, during the second period, it increases to around 28% and then continues to climb gradually. After the 12 periods, Google Trends has up to 60% contribution to dengue incidence variance.

On the other hand, Figure 3C shows that Google Trends variance mainly comes from itself, where dengue incidence only contributes at a rate of approximately 4%. This rate increases in the first four periods (from 3.6% in the first period to 4.2% in the fourth period). Thereafter, the contribution rate from dengue incidence remains around 3.7%. This means that dengue incidence very slightly influences Google Trends in the short term, but does not influence the popularity of Google Trends in the long term.

In summary, it can be seen that Google Trends influences dengue incidence in the long term, but dengue incidence only influences Google Trends in the short term and not in the long term. As presented in the model, dengue incidence is related to not only the popularity of dengue in Google but also its lagged value of up to 1 week.

## Discussion

### Principal Findings

Our results show that there is indeed a causal relationship between dengue popularity in Google Trends and dengue incidence in Bandung. A Granger cointegrated relationship between dengue popularity in Google Trends and dengue incidence in Bandung was noted. This is justified because both data sets were found to be *I*(1), and the residual from the ordinary least squares regression was also found to be stationary.

Based on the ECM, it can be seen that there is a relationship between Google Trends results and dengue incidence. Through Granger analysis, it was seen that Google Trends Granger-causes dengue incidence in Bandung at a lag of 2 and 3 weeks. This was further supported by the impulse response function, where shocks in dengue popularity in Google cause dengue incidence to increase. It was also supported by the variance decomposition, where after 1 week, the contribution from Google Trends to dengue incidence variance increases. Granger analysis also showed that dengue incidence does not Granger-cause its popularity in Google.

The vector ECM also showed that dengue incidence is more responsive to deviations from the long-run equilibrium, since it has a larger value of the speed of adjustment, which is nine times the value for Google Trends.

### Limitations

The results showed a causal relationship between dengue popularity in Google Trends and dengue incidence in Bandung. However, this exact ECM cannot be used for forecasting or early detection owing to the low R-squared values of 0.4128 for the Google Trends equation and 0.1511 for the dengue incidence equation. A further improved model will need to be built for future forecasting.

The results of this study can help provide a more real-time indication of dengue outbreaks in Bandung. Owing to Indonesia’s standard and traditional approach to dengue surveillance, the data of dengue cases have several weaknesses, such as low accuracy and timeliness [20]. In addition, data available from Santo Borromeus Hospital were only from 2008 until 2017. Owing to the limitations of Google Trends, it was preferable to use weekly data with a 5-year period (giving 260 data points) rather than data from 2008 to 2014 on a monthly basis with only 84 data points. Another limitation is that people searching for dengue-related information may not necessarily have the disease, as they could be searching because a relative or friend is ill. Besides, a search in Bandung does not necessarily mean that the intended sick person is in Bandung. The individual could be searching for someone else who is ill in another city.

Our proposed model used strong assumptions, such as the behavior of the use of gadgets and social media in the community, which is quite high, and a good internet signal in the observation area (Bandung in this case). Therefore, it is risky to implement the findings in areas with low internet access.

### Conclusions

Google Trends data may be used as an initial indicator of a dengue outbreak in Bandung. However, further improvements to the ECM need to be made by using more data points to gain more extensive insights.

## Abbreviations

ECM: error correction model
VAR: vector autoregression
